# The President of the Philadelphia County Medical Society

**Published:** 1850-09

**Authors:** 


### THE PRESIDENT OF THE PHILADELPHIA COUNTY MEDICAL SOCIETY.

In the September number of the *Medical Examiner*, Dr. S. Jackson, President of the Philadelphia County Medical Society, treats us to sixteen pages of, what he intends shall be considered a response to our remarks upon his recent lecture on sprinkling streets. We did not receive the *Examiner* in time to respond to Dr. Jackson in the proper department of this number of the Medical Journal, but we shall endeavor to make a reply in the October number. We regret that Dr. Jackson has taken in high dudgeon, remarks of ours that scarcely justify his anger. Because we said that his objections to frozen, and wet pavements, looked very much like murmuring at Providenee, Dr. Jackson infers that we charge him with irreligion, and he takes the matter so much to heart, that in the midst of his sorrows, we are at a loss to know whether he thinks we charged him with atheism or deism. Our argument was a logical one, and manifested neither a religious zeal on our part nor a want of it in Dr. Jackson, but he complains of the blow, from one end of his article to the other.

The question discussed by Dr. Jackson is an important one in hygeine, and we shall endeavor to examine its bearings in a future reply to Dr. Jackson's ill-natured attack.

